# The Impact of Edentulism on Oral and General Health

**DOI:** 10.1155/2013/498305

**Published:** 2013-05-08

**Authors:** Elham Emami, Raphael Freitas de Souza, Marla Kabawat, Jocelyne S. Feine

**Affiliations:** ^1^Département de Dentisterie et de Restauration, Faculté de Médecine Dentaire, Université de Montréal, CP 6128, Succursale Centre-Ville, Montréal, QC, Canada H3C 3J7; ^2^Department of Dental Materials and Prosthodontics, Ribeirão Preto Dental School, University of São Paulo, 14040-904 Ribeirao Preto, SP, Brazil; ^3^Oral Health and Society Research Unit, Faculty of Dentistry, McGill University, Montreal, QC, Canada H3A 0C7; ^4^Department of Epidemiology and Biostatistics and Department of Oncology, Faculty of Medicine, McGill University, Montreal, QC, Canada H3A 1A2

## Abstract

An adequate dentition is of importance for well-being and life quality. Despite advances in preventive dentistry, edentulism is still a major public health problem worldwide. In this narrative review, we provide a perspective on the pathways that link oral to general health. A better understanding of disease indicators is necessary for establishing a solid strategy through an organized oral health care system to prevent and treat this morbid chronic condition.

## 1. Epidemiology of Tooth Loss

Edentulism is a debilitating and irreversible condition and is described as the “final marker of disease burden for oral health” [1]. Although the prevalence of complete tooth loss has declined over the last decade, edentulism remains a major disease worldwide, especially among older adults [2] (Table 1). However, there are intra- and intercountry variations in the prevalence of complete edentulism [3], and direct comparison between national samples is difficult because of the impact of various factors like education, economic circumstances, lifestyle, oral health knowledge and beliefs, and attitudes to dental care [4]. In the United States, the number of edentate individuals is likely to stay stable at 9 million and, according to the most recent information, the prevalence of edentulism amongst adults over 60 years of age was 25% [5]. In 2010, the overall rate of edentulism in Canada was 6.4%, and among adults between 60 and 79 years of age, it was 21.7% [6]. The rate of edentulism tends to vary among different regions within a country. In Canada, there is a wide variation between provinces, from 14% (Quebec) to 5% (Northwest Territories) due to associated factors such as access to fluoridated water and smoking [3]. In Brazil, the wealthier, more industrialized states tend to have lower rates than other parts of the country [7].

Studies show that edentulism is closely associated with socioeconomic factors and is more prevalent in poor populations and in women [3, 16]. In 2003, the ratio of edentulism was 6 times higher in low-income than in higher income Canadian families [3]. Other factors contributing to the prevalence of complete tooth loss are age, education, access to dental care, dentist/population ratios, and insurance coverage [17, 18]. Most edentate people are elders who wear complete dentures in one or both jaws. Studies have demonstrated that denture wearing continues to increase due to the increase in the aging population; a large number of people still depend on removable dentures for oral function [2].

Edentulism can lead directly to impairment, functional limitation, physical, psychological, and social disability, and handicap [19]. Thus, the impact of edentulism on general health should be examined by analyzing the major dimensions of health: physical symptoms and functional capacity, social functioning and perception of well-being. This means that well-quantified endpoints of demographic significance can be used to understand the global burden of this disease. The literature has been reviewed accordingly.

## 2. Impact of Edentulism on Oral Health

### 2.1. Tooth Loss: Modifier of Normal Physiology

Bone loss is an ongoing process following tooth loss [20], affecting the mandible four times more than the maxilla [21]. Edentulism was found to have a significant effect on residual ridge resorption [22], which leads to a reduction in the height of alveolar bone and the size of the denture bearing area. This reduction affects face height and facial appearance, which are altered following total tooth loss [20]. The loss of alveolar bone height and width also leads to substantial changes in the soft-tissue profile, such as protrusion of the mandibular lip and chin [23]. There exists an interpatient variation in these anatomic degenerative changes, and the etiology of these is still unclear. It is believed that a combination of local and systemic factors may be contributors; these include age, gender, duration of edentulism, parafunctional habits, general health, and several diseases [24].

### 2.2. Tooth Loss: Risk Factor for Impaired Mastication

The number of teeth has been chosen as a key determinant of oral function and oral health status [25, 26]. Several studies using different methodologies have demonstrated that an important indicator for masticatory efficiency is the number of functional tooth units [27, 28]. According to a systematic review evaluating the relationship between oral function and dentition, tooth numbers below a minimum of 20 teeth, with nine to 10 pairs of contacting units, are associated with impaired masticatory efficiency, performance, and masticatory ability (an individual's perception of his/her ability to chew) [25].

Although some evidence suggests that reduced oral function in elders is related to muscle atrophy, aging alone has little impact on masticatory performance [29]. Most studies agree that denture wearers have only about one-fifth to one-fourth the bite strength and masticatory force of dentate individuals [30]. Furthermore, complete denture wearers require 7 times more chewing strokes than those with natural dentitions to be able to cut food into half of its original size [31]. Moreover, the thickness of the masseter muscle was found to be decreased in edentulous patients, thus decreasing bite force [32].This may partly explain why individuals wearing complete dentures have difficulty chewing hard foods.

This disability could substantially influence the desire to bite, to chew, and to swallow and could lead to a modification of food choices [33, 34]. As a result, research has consistently demonstrated that tooth loss and dental status have a negative impact on diet and food selection [20, 35].

### 2.3. Tooth Loss: Determinant of Oral Health

Edentulism can be accompanied by functional and sensory deficiencies of the oral mucosa, oral musculature, and the salivary glands. Decreased tissue regeneration and decreased tissue resistance are expected in the edentulous population, which can impair the protective function of the oral mucosa. Associations have been reported between aging, denture use, and oral mucosal disorders, including denture stomatitis, an inflammatory condition of the palatal mucosa seen in complete denture wearers, angular cheilitis, oral candidosis, and traumatic ulcers [36–38]. According to MacEntee et al., the odds of finding hyperplasia, stomatitis, and angular cheilitis increase approximately three-fold in denture wearers [36]. Such disorders could expose the individual to internal and external pathogens, and their prevalence is an important parameter in evaluating the oral health of an elderly population [39, 40]. Although a direct correlation between edentulism and aspiration pneumonia has not been reported, the potential relation between denture plaque and aspiration pneumonia has been discussed in susceptible individuals [41, 42]. Although the majority of oral mucosal conditions in the elderly are benign, some may become malignant, especially if the protective functions of oral mucosa are decreased [37].

Edentulism may induce an oral dyskinesia, defined as abnormal, involuntary, patterned or stereotyped, and purposeless orofacial movements. Several factors, such as ill-fitting and unstable prostheses, oral discomfort, and lack of sensory contacts, have been proposed to explain oral dyskinesia in edentulous individuals, but the exact mechanism is still unclear. Denture wearers may have additional prosthetic problems as a result of soft and hard tissue damage caused by oral dyskinesia [43]. Edentulism is also associated with tardive dyskinesia, a type of dyskinesia occurring among patients chronically treated with antipsychotic drugs [44].

## 3. Impact of Edentulism on General Health

According to several studies, tooth loss can affect general health in several ways as indicated as follows

- lower intake of fruits and vegetables, fiber, and carotene and increased cholesterol and saturated fats, in addition to a higher prevalence of obesity, can increase the risk of cardiovascular diseases and gastrointestinal disorders [14, 45];
- increased rates of chronic inflammatory changes of the gastric mucosa, upper gastrointestinal and pancreatic cancer, and higher rates of peptic or duodenal ulcers [46–48];
- increased risk of noninsulin-dependent diabetes mellitus [49, 50];
- increased risk of electrocardiographic abnormalities, hypertension, heart failure, ischemic heart disease, stroke, and aortic valve sclerosis [46, 51–53]. A study also demonstrated a possible association between complete edentulism and an increased risk of coronary heart disease [54]. Furthermore, a more recent large prospective study concluded that the number of teeth was a dose-dependent predictor to cardiovascular mortality [55];
- decreased daily function, physical activity, and physical domains of health-related quality of life [56, 57];
- increased risk of chronic kidney disease [58];
- association between edentulism and sleep-disordered breathing, including obstructive sleep apnea [59].

Although evidence is accumulating to support a reciprocal relationship between oral and general health [38, 60], the mechanisms linking poor general health and tooth loss are not yet clear. A purported pathway for this association involves deleterious effects of tooth loss on nutrition that, in turn, impacts systemic health [61]. Nutritional factors, especially antioxidants, may decrease following tooth loss and modulate systemic disease by interfering with the inflammatory cascade and preventing carcinogenesis [61]. A study on 83,104 US women [45] showed that diet might partially explain the association between oral health and cardiovascular disease. In this cross-sectional analysis, the edentulous women had dietary intakes associated with an increased rate of cardiovascular disease. These results are supported by a longitudinal analysis on 41,891 adults, which confirms an association between tooth loss and the prevalence of heart diseases [53].

Furthermore, excessive intakes of highly processed high-fat and high-carbohydrate foods contribute to obesity and obesity-related diseases, such as insulin resistance, cardiovascular disease, and hyperlipidemia [62]. However, it should be understood that the nutritional consequences of edentulism are complex due to a plethora of factors that influence food intake and nutritional status, including acute and chronic disease, alterations in the gastrointestinal tract, functional disabilities, chewing problems, psychological and social factors, and lowered socioeconomic status [63, 64].

Several longitudinal, prospective, and cross-sectional studies have supported the association between tooth loss, diet, and nutrition. Impaired dentition imposes dietary restriction and affects food taste, food selection, food preparation and food eating patterns [35, 65–67]. Results of a study by Locker [68] indicated that 39% of edentulous elders were prevented from eating foods they would like to eat, 29% reported a decline in their enjoyment of food, and 14% avoided eating with others. Suboptimal diets may prevent edentulous individuals from meeting recommended dietary allowances and lead to compromised nutritional states, especially in edentulous subjects without dentures [63, 69, 70]. Studies have demonstrated that diet in edentulous subjects consists of food that is low in fiber and high in saturated fat, with a significant lack of intake of high-fiber foods such as breads, fruits, vegetables, and nonstarch polysaccharides (NSP) [35, 63, 65, 67]. Low NSP intakes (>10 g/d) and low fruit and vegetable intakes (>160 g/d) have been reported in edentulous people [71]. Joshipura et al. [72] collected dietary intake data from 49,501 male health professionals and demonstrated that, compared to dentate individuals, edentulous respondents consumed fewer vegetables, less fiber, and less carotene intake, while consuming more cholesterol and saturated fats. These differences were independent of sociodemographic and health behaviour characteristics. Lowe et al. [73] established that total tooth loss was associated with low citrus fruit consumption, low plasma vitamin C levels, and increased amounts of inflammatory reactants, such as plasma C-reactive protein. They also demonstrated increased levels of plasma interleukin-6, fibrinogen, and factor VIII in women. These factors are associated with an increased risk of coronary heart diseases and stroke.

In relation to weight gain, the results of a study carried out by Lee et al. [74] demonstrated that edentulism was associated with a weight gain of >5% in one year. Furthermore, an association between edentulism and obesity was found in several studies [14, 75]. When edentulism was not rehabilitated with complete dentures, it was associated with both underweight and overweight/obesity in an elderly population [76].

Despite this evidence, some findings contradict the association between dentition and nutrition [77, 78]. In a cross-sectional study, Shinkai et al. [77] investigated the influence of dentition status on overall diet quality. The authors concluded that, although individuals with better dentition status had better masticatory performance and bite force, no association was found between dentition status and quality of diet. However, in the same study, they found an association between masticatory variables and intakes of specific dietary components, such as vitamin C and fiber. There also exist some contradicting results regarding the influence of sociodemographic variables on the dentition-nutrition relationship. Findings of Nowjack-Raymer and Sheiham [66] demonstrated that the association between dentition and nutrition was independent of age, sex, raceethnicity, and socioeconomic factors, whereas Lee et al. [74] demonstrated racial-ethnic differences in dietary intake patterns, showing that the food intake of African American edentulous elders was similar to those with teeth. However, Caucasian edentate elders displayed different dietary food patterns than their dentate counterparts. This ethnic difference could be explained by fundamental differences in socioeconomic characteristics of racial groups. African Americans consumed more fat, fewer vegetables, and less fiber than did the Caucasians, irrespective of dental condition [79].

Although diet has been shown to be poorer in edentulous populations, there is still a need for more research about the association between tooth loss and specific changes in nutrient intake. The association between tooth loss and aging [17] may become even more important with the growth of the elder population worldwide. Such an increasing population has higher prevalence of chronic conditions [80] that may be indirectly aggravated by edentulism [73].

Regarding life expectancy of edentulous individuals, tooth loss was found to be associated with the onset of disability and mortality, even after adjusting for confounding factors such as socioeconomic and health behavior factors [81], and one study demonstrated that each tooth that remains in the oral cavity after the age of 70 decreased the risk of mortality over 7 years by 4% [82]. In addition, several studies established an association between edentulism before the age of 65 and an increased risk of earlier death [83]. Also, according to Shimazaki et al. [84], the mortality rate of edentulous elders without dentures was significantly higher than those with 20 or more teeth and, in a large cohort study, an association was found between tooth loss and mortality, in addition to death resulting from gastrointestinal cancer, heart disease, and stroke [46].

## 4. Impact of Edentulism on the Quality of Life

The term “quality of life” is often used as an umbrella term that covers various concepts, that is, health status, function, and life conditions. In general, quality of life (QoL) is defined as an individual's perception of his or her position in life, in the context of the culture and value systems in which they live, and in relation to their goals, expectations, and concerns [85]. Perception of QoL varies among individuals and fluctuates over time for the same person as a result of changes in any of its component parts [86]. QoL is partly affected by a person's oral health. Perceptions of how oral conditions affect daily function and well-being are referred to as oral health-related quality of life (OHQoL) [87–89]. OHQoL has been widely used in clinical studies as an outcome to assess the quality, effectiveness, and efficacy of oral health care [87, 90].

Increasingly, it is recognized that patients' perceptions of their oral health are important in evaluating well-being and determining health care outcomes [91]. The exclusive use of clinical measures has been generally criticized because they provide little insight into the psychosocial aspects of health and do not adequately reflect the health status, functioning, and perceived needs of edentulous and elderly individuals [33, 92, 93]. Edentulism may lead to changes in most of the domains leading to poorer QoL (e.g. impaired mastication, denture trauma, aesthetic concerns, or negative self-perception). Teeth have an important role in facial appearance, speech, and eating ability. There is overwhelming evidence showing the negative effect of edentulism on OHQoL [94, 95]. Edentulism negatively influences not only oral function, but also social life and day-to-day activities [96]. Compromised oral function has been linked to decreased self-esteem and a decline in psychosocial well-being [97]. Edentulous people may avoid participation in social activities because they are embarrassed to speak, smile, or eat in front of others, leading to isolation [98]. Many people develop skills to overcome the limitations of dentures, but some are unable to do so [20]. Fiske et al. [99] demonstrated that denture wearers have decreased self-confidence, premature aging, altered self-image, and altered behaviour in socializing and forming close relationships. On the other hand, dentures could improve oral appearance and social interactions of individuals, which might enhance self-esteem and thus contribute to psychological well-being [90, 100]. Variables, including type of treatment, age, sex, and marital status, could explain the variation in ratings of OHQoL and tooth loss [90].

## 5. Conclusion

Edentulism has a series of deleterious consequences for oral and general health. Oral consequences vary from the well-known residual ridge resorption to an impaired masticatory function, an unhealthy diet, social disability, and poor oral health quality of life. Edentulous individuals are also in greater risk for different systemic diseases and an increase in mortality rate. Therefore, oral health care providers should prevent tooth loss with proper dental education, oral health promotion, and a high level of dental care in an attempt to assure the existence of a physiologic dentition.

## Figures and Tables

**Table 1:** Prevalence of edentulism in the elderly from different countries.

Region or country	Year of survey	Sample size	Age group (years)	Percentage edentulous
United States, data from the National Health and Nutrition Examination Survey [8]	2009-2010	about 5,000	65–74	15%
			≥75	22%
Canada [6]	2007–2009	6,000	20–79	6%
			60–79	22%
Brazil [9]	2002-2003	5,349	65 to 74	54.7%
Mexico [10]	2002-2003	54,638,654	≥18	6.3%
			65–74	25.5%
Valencia, Spain [11]	2006	1,264	65–74	20.7%
Montpellier, France [12]	2004	321	65+	26.9%
Turkey [13]	2004-2005	1,545	65–74	48.0%
Sweden [14]	2002	16,416	55–84	14%
Hungary [15]	2004	4,606	65–74	19.8%
			≥75	38.7%
